# High-resolution mapping of plasmid transcriptomes in different host bacteria

**DOI:** 10.1186/1471-2164-10-12

**Published:** 2009-01-09

**Authors:** Masatoshi Miyakoshi, Hiromi Nishida, Masaki Shintani, Hisakazu Yamane, Hideaki Nojiri

**Affiliations:** 1 Biotechnology Research Center, The University of Tokyo, 1-1-1 Yayoi, Bunkyo-ku, Tokyo 113-8657, Japan; 2 Agricultural Bioinformatics Research Unit, Graduate School of Agricultural and Life Sciences, The University of Tokyo, 1-1-1 Yayoi, Bunkyo-ku, Tokyo 113-8657, Japan; 3 Department of Environmental Life Sciences, Graduate School of Life Sciences, Tohoku University, 2-1-1 Katahira, Sendai 980-8577, Japan

## Abstract

**Background:**

Plasmids are extrachromosomal elements that replicate autonomously, and many can be transmitted between bacterial cells through conjugation. Although the transcription pattern of genes on a plasmid can be altered by a change in host background, the expression range of plasmid genes that will result in phenotypic variation has not been quantitatively investigated.

**Results:**

Using a microarray with evenly tiled probes at a density of 9 bp, we mapped and quantified the transcripts of the carbazole catabolic plasmid pCAR1 in its original host *Pseudomonas resinovorans *CA10 and the transconjugant *P*. *putida *KT2440(pCAR1) during growth on either carbazole or succinate as the sole carbon source. We identified the operons in pCAR1, which consisted of nearly identical transcription units despite the difference in host background during growth on the same carbon source. In accordance with previous studies, the catabolic operons for carbazole degradation were upregulated during growth on carbazole in both hosts. However, our tiling array results also showed that several operons flanking the transfer gene cluster were transcribed at significantly higher levels in the transconjugant than in the original host. The number of transcripts and the positions of the transcription start sites agreed with our quantitative RT-PCR and primer extension results.

**Conclusion:**

Our tiling array results indicate that the levels of transcription for the operons on a plasmid can vary by host background. High-resolution mapping using an unbiased tiling array is a valuable tool for the simultaneous identification and quantification of prokaryotic transcriptomes including polycistronic operons and non-coding RNAs.

## Background

Prokaryotic transcriptomes change not only in response to physiological parameters but also to genetic rearrangements mediated by mobile elements. Plasmids are extrachromosomal genetic elements that replicate autonomously, and many can be transmitted between different strains through conjugation. Plasmids provide benefits to their hosts, such as resistance to antibiotics or degradation of recalcitrant aromatic compounds [1]; however, in several cases, the carriage of a large plasmid results in changes in the transcriptome of the host chromosome [2-4]. Similar to the effects of plasmid carriage on the transcriptional network of the host chromosome, differences in host background can alter the transcription patterns of backbone and accessory genes on a plasmid. Many plasmid backbone genes essential for conjugative transfer, replication initiation, and active partitioning are regulated both autogenously and by host factors [5]. Additionally, a number of plasmid-encoded degradative accessory genes that constitute relatively large catabolic operons [6] are coordinately regulated by specific plasmid-encoded transcriptional regulators and chromosomal regulatory elements [7].

The 199,035-bp completely sequenced catabolic plasmid pCAR1, which was originally isolated from *Pseudomonas resinovorans *CA10, encodes the degradation pathway of carbazole, a nitrogen-containing recalcitrant aromatic compound [8-10]. pCAR1 carries the *car *and *ant *operons, both of which are induced by anthranilate, a carbazole intermediate, and which are under the control of the AraC/XylS family activator AntR [11,12]. pCAR1 has been characterized as a self-transmissible and narrow-host-range plasmid that belongs to incompatibility group P-7 (IncP-7); the conjugative transfer of pCAR1 enables the completely sequenced bacterium *P*. *putida *KT2440 [13] to grow on carbazole as the sole carbon source [14]. Using an expression microarray of the KT2440 chromosome coupled with pCAR1, we previously analyzed the differential expression of both pCAR1 and the KT2440 chromosome during growth on carbazole and succinate, and found that pCAR1 successfully functioned in the host and affected the chromosomal transcriptome [4]. However, we did not address whether and how the plasmid transcriptome in the original host differed from that in the transconjugant; thus, in this study, we focused on the transcriptomes of pCAR1 in the heterologous host bacteria.

Recent increases in microarray feature density have allowed the construction of tiling arrays, which contain overlapping probes that may be used to target any region of a sequenced genome. Transcriptome analyses using unbiased high-density tiling arrays have been used to detect individual exons of a spliced transcript and multifunctional and extensive transcription from both strands of human DNA [15]. As for prokaryotic transcriptomes, high-density tiling arrays aimed at the genome-wide determination of transcription start sites in *E*. *coli *and *Caulobacter crescentus *contain probes targeting intergenic regions [16,17]. In this study, we used an unbiased tiling array with evenly tiled probes at a 9-bp density to identify the entire operonic structure from transcription initiation to termination. The use of a high-resolution tiling array allowed us to visualize the transcriptome of the plasmid independent of the host background. A comparison of plasmid transcriptomes between the original host and transconjugant revealed similarities and differences in the transcription patterns of the plasmid.

## Results and Discussion

### Mapping of single-stranded cDNA using the pCAR1 tiling array

Two pCAR1-harboring strains, the original host *P*. *resinovorans *CA10 and the transconjugant *P*. *putida *KT2440(pCAR1), were grown to the exponential phase using carbazole or succinate as the sole source of carbon. Single-stranded cDNA was synthesized from total culture RNA using random primers. Although the Affymetrix RNA mapping protocol recommends synthesizing second-strand DNA from first-strand cDNA using a DNA polymerase, we hybridized the single-stranded cDNA to the tiling array in order to preserve the strand directionality of the prokaryotic transcriptome.

**Figure 1:**
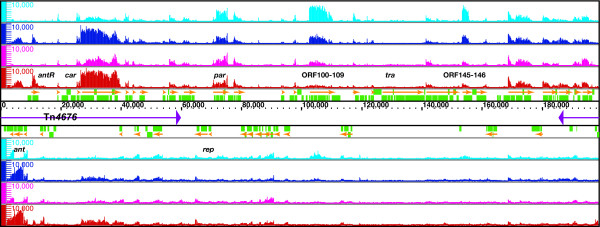
**High-resolution mapping of the pCAR1 transcriptome**. Single-stranded cDNA from KT2440(pCAR1) grown on succinate (cyan) or carbazole (blue) and from CA10 grown on succinate (magenta) or carbazole (red) was mapped on both strands of pCAR1, which are transcribed from left to right (above) and from right to left (below). The identified transcripts and annotated ORFs are indicated by orange arrows and green rectangles, respectively. The scale bars indicate the signal intensities from 0 to 10,000 on a linear scale.

The resulting high-resolution map showed that the pCAR1 transcriptome consisted of identical transcription units regardless of the host background (Figure 1). Using the Integrated Genome Browser (IGB, Affymetrix), we identified a total of 49 transcription units that were transcribed continuously at a signal intensity above 100 (Table 1), including two overlapping operons that were transcribed from multiple initiation sites and three small transcripts identified as non-coding RNAs (ncRNAs). The average length of each transcription unit was 3.1 kb, and 72% (~144.2 kb) of the entire sequence of pCAR1 was transcribed.

**Table 1:** Transcription units of pCAR1 identified by tiling array analysis at 9-bp resolution.

Unit	Direction	Transcription start site	Transcription termination site	Length	Genes or ORFs	KT_SUC sense	KT_SUC antisense	KT_CAR sense	KT_CAR antisense	CA_SUC sense	CA_SUC antisense	CA_CAR sense	CA_CAR antisense
#1	-	4440	3210	1230	*tnpA3*	2164	428	1879	378	1386	236	2339	386
#2	-	7530	4440	3090	*antA-antC*	86	86	4410	1399	136	78	4883	1810
#3	-	8830	7440	1390	*tnpA2*	1153	161	1266	201	929	124	851	100
#4	+	10580	12310	1730	*antR*	54	45	1271	277	52	25	2902	430
#5	-	14440	13310	1130	ORF21	310	110	486	175	513	187	1325	491
#6	+	18860	19730	870	ORF16	678	124	395	141	465	84	405	72
#7	+	25320	26350	1030	*tnpA1*	1450	182	1375	219	1045	115	1001	104
#8A	+	26620	40170	13550	ORF9-*carE*	589	151	4068	646	1444	319	5481	946
#8B	+	27760	40170	12410	*carAa-carE*	645	159	3919	632	1552	358	5488	929
#9	-	40980	39560	1420	ORF37	576	142	658	252	936	182	875	186
#10	+	41500	42530	1030	*tnpA4*	1420	193	1320	217	1103	119	943	109
#11	+	43880	44630	750	ORF40	577	372	623	335	987	351	706	211
#12	-	46080	44190	1890	ORF42-41	898	300	903	460	653	234	637	173
#13	+	46110	48370	2260	*tnpRaAa*	296	183	580	257	168	82	129	76
#14	-	53780	50550	3230	*tnpS-*ORF47	839	212	780	277	494	109	409	72
#15	+	53890	55400	1510	*tnpT*	261	94	458	145	143	56	137	46
#16	+	56180	58400	2220	ORF53-54	719	216	582	222	1073	255	789	150
#17	+	60210	64660	4450	*klaA-*ORF59	356	120	269	105	353	130	368	119
#18	-	68940	64440	4500	ORF61-ORF60	217	101	214	93	246	128	267	101
#19	-	70120	69120	1000	*repA*	347	67	235	65	362	82	372	85
#20A	+	70060	75580	5520	*parW-*ORF67a	2441	364	1316	175	1372	255	1257	202
#20B	+	71770	75580	3810	*parA-*ORF67a	3199	506	1713	231	1924	319	1562	221
#21	+	77490	80400	2910	*pmr-*ORF72	1163	269	769	184	752	198	728	181
#22	-	81820	79480	2340	ORF74-73	318	174	271	183	388	169	427	150
#23	-	84010	82170	1840	ORF77-75	401	88	301	76	294	78	411	87
#24	+	85720	85870	150	ncRNA1	1301	543	1168	470	403	150	1283	114
#25	-	86510	84550	1960	ORF81-79	249	61	291	72	196	48	262	60
#26	-	90880	87680	3200	ORF89-85	880	158	406	93	1051	211	620	128
#27	-	92880	91500	1380	ORF91	283	93	123	94	207	75	146	80
#28	+	93360	94230	870	ORF92	569	100	357	64	366	86	479	78
#29	-	95580	94380	1200	ORF93	541	187	404	149	482	152	559	169
#30	+	97170	98100	930	ORF95a	848	146	627	121	607	110	587	107
#31	+	102560	110380	7820	ORF100-109	4564	554	1588	169	667	80	443	59
#32	-	117040	113160	3880	ORF115-111	264	76	129	58	302	93	165	70
#33	+	117170	119180	2010	*ssb-recT*	719	103	249	33	208	34	114	19
#34	-	119470	119330	140	ncRNA2	806	725	656	545	242	254	237	168
#35	+	121840	121950	110	ncRNA3	1847	573	1562	472	581	125	694	129
#36	+	123060	124110	1050	ORF120	458	60	598	62	189	39	400	47
#37	+	127040	140790	13750	*traG-*ORF131	125	46	73	44	191	70	150	63
#38	+	141370	152460	11090	ORF134-143	252	150	204	188	235	120	187	118
#39	+	153440	155620	2180	ORF145-146	4464	645	2126	311	897	149	801	115
#40	+	155840	161570	5730	*traF-trhG*	181	134	135	212	180	103	120	107
#41	-	165130	161590	3540	ORF154-150	193	60	180	102	217	63	222	47
#42	+	168730	176860	8130	ORF159-167	1295	263	1296	272	1082	214	1148	181
#43	-	180180	179390	790	*tnpRb*	283	68	439	113	144	28	223	30
#44	+	180340	185550	5210	ORF170-175	717	165	899	218	492	106	765	153
#45	+	185870	189340	3470	*tnpAcC*	310	307	1004	536	76	93	853	290
#46	+	190420	192860	2440	ORF179-181	746	489	640	349	664	379	608	308
#47	+	193300	195590	2290	ORF182-184	1451	227	1167	208	1942	330	1537	207

The transcription units of pCAR1 and their median signal intensities on both the sense and antisense strands are indicated; KT_SUC, KT2440(pCAR1) grown on succinate; KT_CAR, KT2440(pCAR1) grown on carbazole; CA_SUC, CA10 grown on succinate; CA_CAR, CA10 grown on carbazole. The positions of the transcription start and stop sites correspond to the pCAR1 nucleotide sequence [GenBank:AB088420].

**Figure 2:**
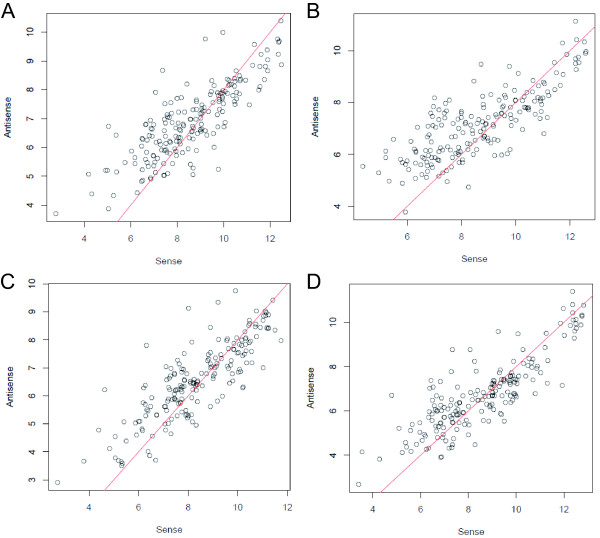
**Scatterplots of the median signal intensities (log_2_) within a sense region and its antisense region**. (A) KT2440(pCAR1) grown on succinate; *r *= 0.82 (n = 190). (B) KT2440(pCAR1) grown on carbazole; *r *= 0.79 (n = 190). (C) CA10 grown on succinate; *r *= 0.83 (n = 190). (D) CA10 grown on carbazole; *r *= 0.84 (n = 190). Red lines, y = x-2.

**Figure 3:**
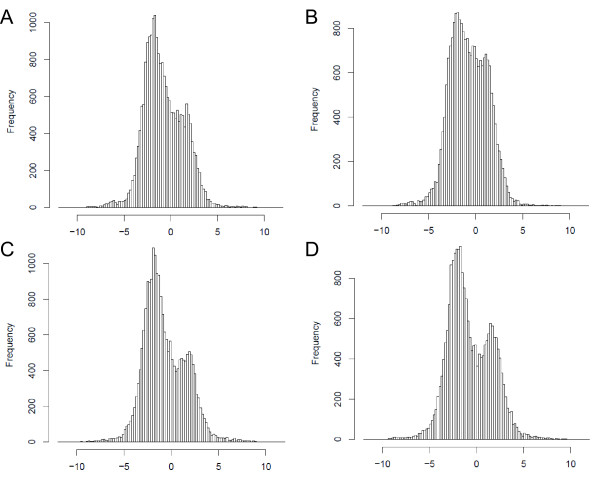
**Histograms showing the log_2 _ratio of the signal intensities for the sense and antisense strands**. The frequency of the signal intensity ratios for a total of 21,875 probe pairs was plotted. All four histograms have two peaks (at 2 and -2), indicating that most of the ratios were 4 or 0.25. (A) KT2440(pCAR1) grown on succinate. (B) KT2440(pCAR1) grown on carbazole. (C) CA10 grown on succinate. (D) CA10 grown on carbazole.

Unexpectedly, we observed numerous signals on the antisense strand in the coding regions. These signals likely represent experimental artifacts generated by secondary mispriming during reverse transcription using random primers [18,19] since no signal was detect on the antisense strand by hybridization of cDNA synthesized using strand-specific primers (data not shown). To estimate how much of the single-stranded cDNA synthesized using random primers contained these artifacts, the median signal intensities were calculated from probes located within the pCAR1 genes. The correlation coefficients between the sense and antisense values on a log_2 _scale were 0.79–0.84 (Figure 2). The plots were approximated to the line y = x-2, indicating that the intensity of the sense signals was approximately four times higher than that of the antisense signals. In addition, the log_2 _ratios of the signal intensities for each probe on one strand to those on the other strand were frequently 2 and -2 (Figure 3), suggesting that ~20% of the signals detected on the antisense strand in the coding regions represent artifacts. Therefore, we subsequently analyzed only the sense signal intensities.

**Figure 4:**
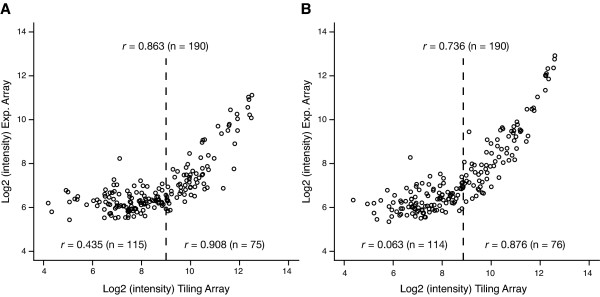
**Comparison of the dynamic range between the tiling array and the expression array platforms**. The plots represent the median signal intensities (log_2_) of 190 pCAR1 genes calculated from the tiling and the expression arrays. The correlation coefficients are for the signal intensities of the overall genes, highly transcribed genes (> 2^9 ^in the tiling array), and weakly transcribed genes (< 2^9^).

To estimate the sensitivity of the tiling array, the median signal intensities of the pCAR1 genes in KT2440(pCAR1) were compared to those calculated as part of our previous expression array analysis [4]. Overall, the signal intensities of the pCAR1 genes, especially the frequently transcribed genes, exhibited a strong correlation between the two microarray platforms (Figure 4). However, a significant decrease in the Pearson correlation coefficient was detected in the low-signal-intensity plots, which were distributed more widely in the tiling array analysis than in the expression array analysis, indicating that tiling arrays are superior in terms of sensitivity to traditional gene-centered microarrays.

### Transcription pattern of the pCAR1 accessory genes

**Figure 5:**
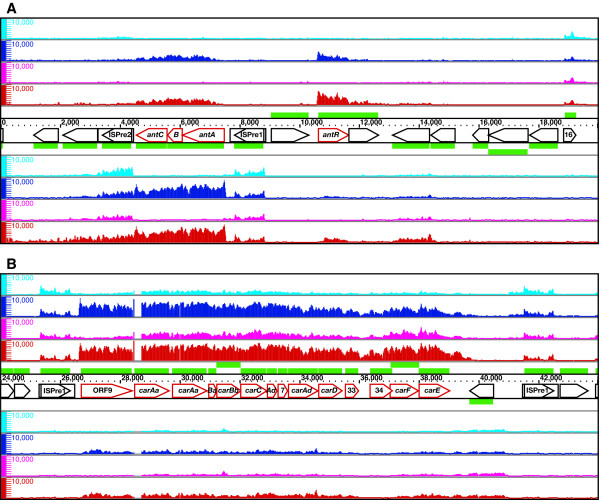
**High-resolution mapping of the catabolic operons induced during growth on carbazole, the *ant *operon and *antR *(A), and the *car *operon (B)**. Single-stranded cDNA from KT2440(pCAR1) grown on succinate (cyan) or carbazole (blue) and from CA10 grown on succinate (magenta) or carbazole (red) was mapped on both strands of pCAR1, which are transcribed from left to right (above) and from right to left (below). Pentagons represent the pCAR1 genes and their transcriptional directions; the gene names or ORF numbers are indicated therein. The scale bars indicate the signal intensities from 0 to 10,000 on a linear scale. pCAR1 carries duplicate *carAa *genes [9]; several probes within the first copy of *carAa *were deleted by the Affymetrix Custom Array Design Program.

pCAR1 carries a 72.8-kb class II transposon, Tn*4676 *(1–59, 548 and 185, 774–199, 035 on pCAR1), which confers the ability to degrade carbazole [20]. Within Tn*4676*, the *ant *operon is transcribed from the inducible promoter P_ant _[11], whereas the *car *operon is transcribed from the inducible promoter P_ant _upstream of open reading frame (ORF) 9 and additionally from the constitutive promoter P_carAa _within the coding region of ORF9 [12]. The AraC/XylS family transcriptional regulator AntR, which is encoded on Tn*4676*, activates the P_ant _promoter in response to anthranilate, an intermediate of the carbazole degradation pathway. The transcription of *antR *originates from an RpoN-dependent promoter and is induced during growth on carbazole [4]. As expected, our tiling array results indicated that growth on carbazole strongly induced these catabolic operons in both hosts compared to growth on succinate (Figure 5A and 5B). The 3.1- and 1.7-kb transcripts of *antABC *and *antR *were significantly induced during growth on carbazole (Figure 5A). The ~13-kb transcript of the *car *operon was also detected; it was induced during growth on carbazole and constitutively produced during growth on succinate (Figure 5B). Intriguingly, the level of transcription of the lower *car *operon, which is composed of *carFE*, was apparently higher in CA10 than in KT2440(pCAR1) regardless of the carbon source. The transcription start sites of *antA*, *antR*, and ORF9 indicated by our tiling array (Figure 5A and 5B) agreed with those identified through primer extension analysis [4,11]. Although our previous data showed constitutive transcription of the *car *operon from 385-bp upstream of *carAa *[12], our tiling array analysis detected only slightly constitutive transcription in both strains during growth on succinate and could not identify the transcription start site (Figure 5B).

**Figure 6:**
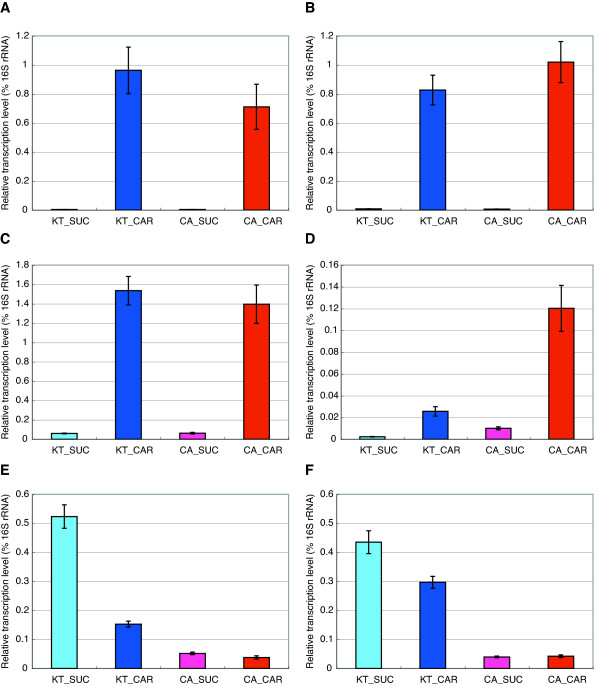
**Quantification of *antA *(A), *antR *(B), ORF9 (C), *carF *(D), ORF100 (E), and ORF145 (F) transcription**. Quantitative RT-PCR was performed using single-stranded cDNA from KT2440(pCAR1) grown on succinate (cyan) or carbazole (blue) and from CA10 grown on succinate (magenta) or carbazole (red). The relative amount of each pCAR1 transcript to 16S rRNA (%) is indicated.

To verify the transcription levels estimated by our tiling array analysis, we performed quantitative RT-PCR and calculated the relative abundance of the pCAR1 mRNAs normalized by 16S rRNA. During growth on carbazole, the transcription of *antA *and *antR *was significantly increased by ~200- and ~100-fold to the same level in both hosts (Figure 6A and 6B). The transcription of the upper *car *operon, which originates from the dual promoters, was also upregulated by ~20-fold with a negligible difference between the hosts (Figure 6C). In contrast, the transcription of the lower *car *operon was significantly higher in CA10 than in KT2440(pCAR1) (Figure 6D). These results suggest that the *car *operon possesses additional promoters that are preferentially activated in the original host.

Tn*4676 *encodes the transposase TnpAc and its repressor TnpC at one end and the cointegrate resolvase TnpST at the other. The transcription of *tnpAc *was induced during growth on carbazole in both hosts, consistent with previous microarray results [4], but the basal level of transcription for *tnpAc *was higher in KT2440(pCAR1) than in CA10 during growth on succinate (Table 1). The levels of the divergent *tnpS *and *tnpT *transcripts were unaffected by the growth conditions and were slightly higher in KT2440(pCAR1) (Table 1). It is noteworthy that Tn*4676 *contains three identical ISPre1 insertion sequences, two of which are located on either end of the *car *operon, with the third located upstream of the *ant *operon [10]. Our tiling array data revealed nearly identical transcription patterns within these insertion sequences (Figure 5A and 5B). This indicates the equivalent detection sensitivities of the contiguous probes, because transcripts of ISPre1 were detected equally on both strands using different probes shifted by several nucleotides.

### Transcription pattern of IncP-7 plasmid backbone

**Figure 7:**
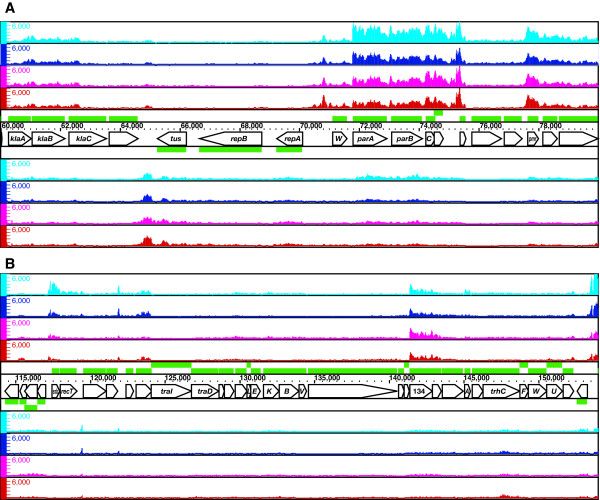
**High-resolution mapping of the IncP-7 plasmid backbone, *rep *and *par *(A) and *tra *(B)**. Single-stranded cDNA from KT2440(pCAR1) grown on succinate (cyan) or carbazole (blue) and from CA10 grown on succinate (magenta) or carbazole (red) was mapped on both strands of pCAR1, which are transcribed from left to right (above) and from right to left (below). Pentagons represent the pCAR1 genes and their transcriptional directions; the gene names or ORF numbers are indicated therein. The scale bars indicate the signal intensities from 0 to 6,000 on a linear scale.

The pCAR1 plasmid contains divergent *repA *and *parWABC *genes for its replication and active partitioning, which are conserved among IncP-7 plasmids [10,21,22]. Our tiling array analysis showed that *repA *was transcribed at a low level and that the level of transcription was unaffected by the host background or carbon source (Figure 7A and Table 1). The transcription of the *par *gene cluster originated from *parW *and *parA*, although the promoter activity of *parA *was much higher than that of *parW *(Figure 7A).

**Figure 8:**
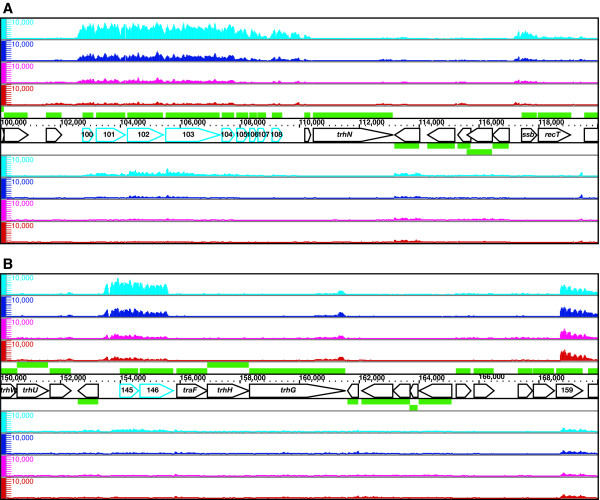
**High-resolution mapping of ORF100-108 (A) and ORF145-146 (B), differentially transcribed operons between KT2440(pCAR1) and CA10**. Single-stranded cDNA from KT2440(pCAR1) grown on succinate (cyan) or carbazole (blue) and from CA10 grown on succinate (magenta) or carbazole (red) was mapped on both strands of pCAR1, which are transcribed from left to right (above) and from right to left (below). Pentagons represent the pCAR1 genes and their transcriptional directions; the gene names or ORF numbers are indicated therein. The scale bars indicate the signal intensities from 0 to 10,000 on a linear scale.

A large gene cluster encoding the conjugative transfer apparatus of IncP-7 plasmids has been reported only for the self-transmissible plasmid pCAR1 [12]. The host range and conjugative transfer frequency of pCAR1 depends on the recipient and donor species; pCAR1 efficiently transfers between species with the same genetic background [23]. High-resolution mapping revealed that the overall transfer gene cluster was transcribed at a low level (Figure 7B); however, two operons flanking the transfer gene cluster (from ORF100 to ORF109 and from ORF145 to ORF146, respectively) were transcribed at significantly higher levels in the transconjugant strain KT2440(pCAR1) than in the original host strain CA10 (Figure 8A and 8B).

**Figure 9:**
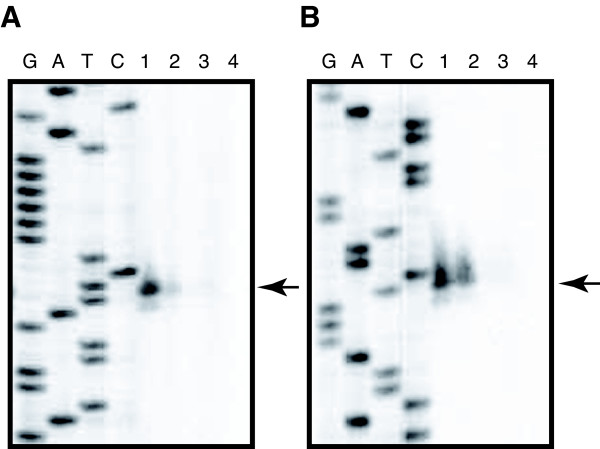
**Mapping of the transcription start sites for ORF100 (A) and ORF145 (B)**. Primer extension was performed using equal amounts of total RNA from KT2440(pCAR1) grown on succinate (lane 1) or carbazole (lane 2) and from CA10 grown on succinate (lane 3) or carbazole (lane 4). Lanes G, A, T, and C indicate the sequence ladder obtained using the same primer.

Quantitative RT-PCR verified that the transcription levels of ORF100 and ORF145 were significantly higher in KT2440(pCAR1) than in CA10 and during growth on succinate than on carbazole (Figure 6E and 6F). To characterize the promoters of ORF100 and ORF145, we performed a primer extension analysis using the same total RNA used in our tiling array analysis. The transcription start sites were mapped to 141-nt and 522-nt upstream of the translation start sites of ORF100 and ORF145, respectively (Figure 9A and 9B), in agreement with our tiling array data. The signal intensities of the primer extension products also corresponded to our quantitative RT-PCR results. However, we were unable to identify a consensus motif between the promoter regions of ORF100 and ORF145. These results suggest that the two operons are regulated by different mechanisms in the two hosts.

A search of the ACLAME database [24] indicated that the genes within ORF100-103 are conserved in the IncH plasmid R478 [25], the IncT plasmid Rts1 [26], and the integrating conjugative elements SXT [27] and R391 [28]. ORF101 encodes a MoxR-like AAA+ ATPase with a C-terminal CbbQ domain that is associated with various cellular activities [29]. Many genes in the AAA+ family have been found in close proximity to genes that encode proteins with von Willebrand factor type A (VWA) domains [29], and we found that ORF103 actually encodes a VWA protein. The VWA domain is a well-studied domain associated with cell adhesion, extracellular matrix proteins, and integrin receptors [30]. ORF145 and ORF146, which encode a putative nickase and primase, respectively, are located upstream of the *traF*-*trhH*-*trhG *gene cluster, which is involved in pilus assembly [23]; however, they were transcribed separately. Our high-resolution map showed a significant decrease in the transcription of ORF146 at approximately nucleotide position 155,630 (Figure 8B), raising the possibility that ORF146 is dysfunctional or at least rarely transcribed. Since the conjugative host range and transfer frequency of pCAR1 are thought to depend on the donor strain [23], these findings suggest that ORF100-109 and ORF145-146 are involved in the conjugative transfer of pCAR1 and that the transcription levels of these operons in donor cells may affect the behavior of pCAR1.

## Conclusion

Using a tiling array, we identified the complete transcriptome of pCAR1 and demonstrated that the level of transcription of several operons was host-dependent. Many of the genes carried on bacterial plasmids have not been adequately annotated or functionally characterized; thus, functional genomic approaches are necessary to identify novel genes with important roles in the relationship between plasmids and their hosts. This study demonstrates that the plasmid transcriptome is affected by the host background, while recent studies have indicated that the carriage of a large plasmid results in a change in the transcriptome of the host chromosome [2-4]. Therefore, we conclude that conjugative transfer results in bidirectional alterations in the transcriptional networks of the plasmid and host chromosome.

The unbiased nature of tiling arrays allows the simultaneous identification and quantification of the prokaryotic transcriptome including polycistronic operons and non-coding RNAs. The transcription start sites identified by tiling array analysis may be verified by primer extension analysis. The number of transcripts indicated by quantitative RT-PCR is comparable among different growth conditions and genetic backgrounds. Tiling array technology is a powerful tool for the analysis of prokaryotic transcriptomes, and it may be used to determine the complete operonic structure of a bacterial genome.

## Methods

### Bacterial strains and growth conditions

*Pseudomonas resinovorans *CA10 [8] and *P. putida *KT2440(pCAR1) [14] were grown in Luria broth and nitrogen-plus mineral medium-4 (NMM-4) as described previously [4]. For RNA extraction, 100 ml of NMM-4 supplemented with 1.0 mg/ml of carbazole or sodium succinate were inoculated with cells from an overnight culture in Luria broth to obtain an initial optical density at 600 nm (OD600) of 0.05. The cells were grown at 30°C on a rotary shaker set at 120 rpm with monitoring of the OD600 or the number of colony-forming units (CFU)/ml to the exponential phase.

### Tiling array design

A high-density oligonucleotide microarray covering all 199,035-bp of the pCAR1 plasmid [GenBank:AB088420] was designed using the CustomExpress Array Program (Affymetrix, Santa Clara, CA). The array contained a total of 88,460 probes consisting of pairs of 25-mer perfect match (PM) probes overlapping at 9-nt offsets, and corresponding mismatch (MM) probes with a one-base mismatch at the thirteenth nucleotide.

### RNA preparation, labeling and hybridization

Two biological replicates were independently prepared. Approximately 1 × 10^9 ^cells of each strain at the exponential phase were mixed with RNA Protect Bacteria Reagent (Qiagen, Valencia, CA) as recommended by the manufacturer. Total RNA was extracted using NucleoSpin RNA II (Macherey-Nagel GmbH & Co. KG, Düren, Germany). The eluted RNA was treated with RQ1 RNase-free DNase (Promega, Madison, WI) at 37°C for 30 min. Following DNase inactivation by the addition of the provided stop reagent and subsequent incubation at 65°C for 10 min, the total RNA was repurified using NucleoSpin RNA Clean-Up (Macherey-Nagel).

Single-stranded cDNA was synthesized in 60 μl of 1× First Strand Buffer (Invitrogen, Carlsbad, CA) containing 12 μg of total RNA, 750 ng of random primers (Invitrogen), 1,500 U of SuperScript II (Invitrogen), 60 U of RNaseOUT (Invitrogen), 10 mM DTT (Invitrogen), 0.5 mM dATP, 0.5 mM dCTP, 0.5 mM dGTP, 0.4 mM dTTP, and 0.1 mM dUTP (Roche Applied Science, Mannheim, Germany). After denaturation of the RNA and random primers at 70°C for 10 min and annealing at 25°C for 10 min, the remaining reagents were added, and the reaction mixture was incubated at 25°C for 10 min, 37°C for 60 min, 42°C for 60 min, and 70°C for 10 min. Following cDNA synthesis, the template RNA was degraded with one-third volume of 1N NaOH at 65°C for 30 min; one-third volume of 1N HCl was added to neutralize the reaction mixture prior to cleanup. The cDNA was purified using a QIAquick PCR Purification Kit (Qiagen).

The cDNA was fragmented and labeled using a GeneChip WT Double-Stranded DNA Terminal Labeling Kit (Affymetrix). The purified single-stranded cDNA (~5 μg) was fragmented in 48 μl of 1× cDNA Fragmentation Buffer containing 15 U of Uracil DNA Glycosylase (UDG) and 225 U of Apurinic/Apyrimidinic Endonuclease 1 (APE1) at 37°C for 60 min followed by incubation at 93°C for 2 min; the majority of the fragmented cDNA was 50–200 bp in length. The fragmented cDNA was labeled in 60 μl of 1× TdT Buffer containing 60 U of Terminal Deoxynucleotidyl Transferase (TdT) and 0.083 mM GeneChip DNA Labeling Reagent at 37°C for 60 min followed by incubation at 70°C for 2 min.

The labeled cDNA was mixed with 50 pM control oligonucleotide B2 (Affymetrix), 1× Hybridization Mix (Affymetrix), and 7% DMSO in a total volume of 200 μl and denatured at 95°C for 5 min. For each array, 130 μl of the hybridization cocktail were hybridized at 45°C for 16 h with a rotation rate of 60 rpm using a GeneChip Hybridization Oven 640 (Affymetrix). The chips were then washed and stained using a Hybridization, Wash, and Stain Kit (Affymetrix) according to the FlexFS450-0002 protocol for GeneChip Fluidics station 450 (Affymetrix). Signals were detected using GeneChip Scanner 3000 7G (Affymetrix).

### Tiling array data analysis

The signal intensity for each probe was computed using Affymetrix Tiling Analysis Software v1.1 (TAS), which uses non-parametric quantile normalization and a Hodges-Lehmann estimator for fold-enrichment (Affymetrix Tiling Array Software v1.1 User's Guide). The intensities were linearly scaled so that the median was 100. The PM and MM intensity pairs were mapped to the plasmid genome. For each position to which a probe pair was mapped, a dataset was generated consisting of all pairs mapping within a window of ± 30 bp, which defines the number of bases extending from the position being analyzed so that every probe in the 61-bp region was included in the signal and *p-*value analysis. The pseudomedian was calculated using a sliding window across the genome as an estimate of the signal intensity per probe position. The significance of the signal intensity was calculated with *p *< 10^-3 ^taken as the threshold.

The intensities from two independent CEL files were analyzed by the MVA plot of TAS; the correlation coefficients between the biological replicates were > 0.95. Two CEL files from independent replicates were converted into BAR files using two separate BPMAP files for the forward and reverse strands. The data were visualized using the IGB package (Affymetrix). We used the median signal intensities of the interior probes as an indicator of the expression level of each gene.

### Quantitative RT-PCR

Quantitative RT-PCR was performed using the ABI 7300 Real-Time PCR System (Applied Biosystems, Foster City, CA) as described previously [4]. The primers used for quantitative RT-PCR (Additional file 1) were designed using the Primer3 program [31]. All products were between 100 and 150 bp in length. For normalization, 16S rRNA was used as an internal standard. The univ16S-F and univ16S-R primer set used to measure the transcription of 16S rRNA was designed based on the 16S rRNA sequences from *P*. *putida *KT2440 [GenBank:AE015451] and *P*. *resinovorans *CA10 [GenBank:AB047273]. All reactions were performed a minimum of three times, and the data were normalized using the average of the internal standard.

### Primer extension

Primer extension analysis was performed using a Li-Cor model 4200L-2 auto-DNA sequencer as described previously [4]. We used the IRD800-labeled primer ORF100-R2 5'-AGGATTCGAGTTCACGGGTA-3' or ORF145-R3 5'-ACGCGGTCAGCACTTTAACT-3' (Aloka, Ltd., Tokyo, Japan), which anneals to the region from +57 to +76 of ORF100 or +353 to +372 of ORF145 relative to the annotated translation start site. A sequence ladder was obtained using the same primer and the appropriate cover clone of pCAR1 [10] as template.

### Accession number

The data were deposited in NCBI's Gene Expression Omnibus (GEO, ) and are accessible through GEO Series accession number GSE10862.

## Authors' contributions

MM conceived the study, carried out the experiments, and drafted the manuscript. MM, HNi, and MS analyzed the data. HNi, MS, HY, and HNo assisted in the preparation of the manuscript. All authors read and approved the final manuscript.

## Supplementary Material

Additional file 1 **Primers used for quantitative RT-PCR. Nucleotide sequences of primers used for quantitative RT-PCR.** Click here for file
