# Supplementary material for: High-resolution mapping of plasmid transcriptomes in different host bacteria
Source: BMC Genomics. 2009 Jan 9;10:12. doi: 10.1186/1471-2164-10-12 (PMC2642839; doi:10.1186/1471-2164-10-12)
Supplement: Additional file 1 — Primers used for quantitative RT-PCR. Nucleotide sequences of primers used for quantitative RT-PCR. [file 1471-2164-10-12-S1.doc]

Supplementary Table 1. Primers used for quantitative RT-PCR.

| Primer | Nucleotide sequence (5’ to 3’) |
| --- | --- |
| univ16S-F | ACACGGTCCAGACTCCTACG |
| univ16S-R | TACTGCCCTTCCTCCCAACT |
| antA-F | TGGAACAAGACCGAGATCAA |
| antA-R | CGGAGACGTTGAAGAAGTCC |
| antR-F | CCTTGATGTGCATGTTCGAG |
| antR-R | GGGTACTGGGTCTTGAGCAG |
| ORF9-F | GTGAGAAGGTCTGGGCAATC |
| ORF9-R | GGCCTATTCTCAGCGTCTTG |
| carF-F | CGAGGGACTTCTTGAGATGC |
| carF-R | ATAACGCGCACACCTTTTTC |
| ORF100-F | CTTCGGGGTTCGTGATCTTA |
| ORF100-R | TGCCAGATTTCTTTGATTCA |
| ORF145-F | CGCATCAAGAAACAGCTCAA |
| ORF145-R | GATAATGGGCGAAACCTGAA |
